# Influence of comorbidities on functional outcomes in patients with surgically treated fragility hip fractures: a retrospective cohort study

**DOI:** 10.1186/s12877-021-02227-5

**Published:** 2021-04-28

**Authors:** Soo Hoon Yoon, Bo Ryun Kim, Sang Yoon Lee, Jaewon Beom, Jun Hwan Choi, Jae-Young Lim

**Affiliations:** 1 Department of Physical Medicine and Rehabilitation, Korea University Anam Hospital, Korea University College of Medicine, 73 Goryeodae-ro, Seongbuk-gu, Seoul, 02841 Republic of Korea; 2 Department of Rehabilitation Medicine, Seoul National University Boramae Medical Center, Seoul, Republic of Korea; 3 Department of Rehabilitation Medicine, Seoul National University Bundang Hospital, Seoul National University College of Medicine, Seongnam-si, Gyeonggi-do Republic of Korea; 4 Department of Rehabilitation Medicine, Regional Rheumatoid and Degenerative Arthritis Center, Jeju National University Hospital, Jeju National University College of Medicine, Jeju, Republic of Korea

**Keywords:** Frail older adults, Hip fractures, Comorbidity, Recovery of function, Rehabilitation

## Abstract

**Background:**

The incidence and number of fragility hip fractures are gradually increasing, resulting in a wide consumption of medical resources. Various factors affecting functional recovery in patients with fragility hip fractures are known, and comorbid diseases are one of them. The purpose of this study is to determine the effect of comorbidities on functional outcomes in patients surgically treated for fragility hip fractures, thereby contributing to the efficient distribution of medical resources.

**Methods:**

This was a retrospective cohort study performed in the three tertiary rehabilitation facilities. A total of 211 patients (50 men and 161 women; average age 81.6 ± 6.7 years) who had undergone surgery for fragility hip fractures were followed up from immediately after transfer to the Department of Rehabilitation Medicine to 6 months postoperatively. Comorbidities referred to a summary of the following conditions: hypertension, diabetes mellitus, chronic liver disease, dementia, cerebrovascular accident, and osteoporosis. Functional outcomes included Koval’s grade, Functional Ambulatory Category (FAC), Functional Independence Measure (FIM)-locomotion, Modified Rivermead Mobility Index, Berg Balance Scale (BBS), 4-Meter Walking speed Test (4MWT), the Korean version of the Mini-Mental State Examination(K-MMSE), Geriatric Depression Scale (GDS), EuroQol Five-Dimension (EQ-5D) questionnaire, the Korean version of the Modified Barthel Index (K-MBI), the Korean version of the Instrumental Activities of Daily Living (K-IADL), and Korean version of Fatigue, Resistance, Ambulation, Illnesses, and Loss of weight scale (K-FRAIL). For all tests, each patient was assessed immediately after transfer and 6 months post-surgery.

**Results:**

Multivariate linear regression analyses adjusted for age, sex, the initial variable of the functional outcomes, and comorbidities revealed that dementia had a significant negative impact on Koval’s grade and K-FRAIL 6 months postoperatively. Diabetes mellitus had a significant negative impact on the FAC, GDS, EQ-5D, K-IADL, and K-FRAIL 6 months postoperatively. Patients with osteoporosis showed a significant negative outcome of FIM-locomotion 6 months postoperatively. A cerebrovascular accident revealed a significant negative impact on the BBS 6 months postoperatively. In addition, hypertension led to significantly less favorable outcomes of the K-FRAIL 6 months postoperatively.

**Conclusions:**

This study confirmed that comorbidities, particularly dementia and diabetes mellitus, significantly influence functional outcomes 6 months after fragility hip fracture surgeries.

## Background

Fragility hip fractures are defined as fractures caused by low-energy trauma, such as a fall from a standing height or less, or no identifiable trauma [1]. Because of the rapid aging of the South Korean population, the incidence and number of fragility hip fractures are gradually increasing [2]. Fragility hip fractures are associated with a high impact on mortality and disability [3]. Therefore, fragility fractures lead to the widespread use of medical resources and high costs of care [4].

Patients with fragility hip fractures often undergo surgery according to the surgeon’s decision [5]. Following surgery, the patients receive comprehensive rehabilitation treatment to improve functional outcomes, such as self-care training, fall prevention, physical therapy, occupational therapy, nutritional support, psychiatric evaluation, postoperative management, and environmental modification [5].

Numerous studies reporting functional recovery after hip fractures have demonstrated that only 40–70% of patients recover the performance of basic living activities and only 40–60% of patients recover their gait level before the fracture [3]. In addition, the maximum functional recovery after hip fractures is known to occur during the first 6 months after fractures [6]. Therefore, identifying the factors that affect functional recovery for 6 months after a hip fracture surgery is important. Various factors (ie, age, pre-fracture functional status, pre-existing comorbidity, fracture site, type of surgery, delay in operation, the functional level at discharge, or malnutrition) affect postoperative functional outcomes in patients with fragility hip fractures [7]. Many studies have been conducted on the relationship between postoperative functional outcomes and these factors [8–10]. Various comorbidities (eg, osteoporosis, sarcopenia, cognitive impairment, etc.) have been shown to negatively affect functional outcomes [9, 11–13]. Gonzalez-Zabaleta et al. [9] transformed comorbidities with the Charlson comorbidity index score and reported that comorbidities act as predictors of mobility and mortality after hip fractures. Di Monaco et al. [11] found that patients with hip fractures with high handgrip power before rehabilitation have a high Barthel Index score at the 6-month follow-up period. In addition, Feng et al. [12] demonstrated that cognitive impairment adversely affects functional outcomes in patients with hip fractures using the Modified Barthel Index. However, these previous studies did not examine various comorbidities at once or did not evaluate many functional outcome tools. Therefore, the aim of this study was to determine the influence of comorbidities on functional outcomes of patients surgically treated for fragility hip fractures and to summarize the predictors of poor functional outcomes in patients with fragility hip fractures. Understanding the association between pre-existing comorbidities and postoperative functional outcomes may improve decision-making with regard to postoperative evaluation and/or rehabilitation strategies.

## Methods

### Study design

This was a retrospective cohort study performed in three tertiary rehabilitation facilities as part of the fragility fracture integrated rehabilitation management clinical trial [14]. This study was approved by the Institutional Review Board of OO University Hospital (IRB no. 2020AN0489) and was conducted in accordance with the principles of the Declaration of Helsinki.

### Participants

Between March 2017 and February 2019, patients who had undergone surgery for a fragility hip fracture and were transferred to the Department of Rehabilitation Medicine were enrolled at Seoul National University Bundang Hospital, Chung-Ang University Hospital, and Jeju National University Hospital. A total of 211 patients who met the following inclusion criteria were recruited: (1) an acute unilateral hip fracture (femoral neck, intertrochanteric, subtrochanteric); (2) age ≥ 65 years; and (3) having undergone a successful hip surgery, such as closed reduction internal fixation, total hip replacement, and bipolar hemiarthroplasty. Patients were excluded if they had a history of a neurodegenerative disease or unstable cardiorespiratory status; if they had undergone surgery for an infection, arthritis, avascular necrosis, loosening of implants, femoral shaft fractures, acetabular fractures, isolated fractures of the greater or lesser tuberosity, a pathologic fracture caused by a tumor, combined multiple fractures, revision surgery, or severe cognitive dysfunction (obey command ≤1 step), or if they declined to participate in the clinical trial.

### Pre-existing comorbidities

A chart review of all patients was conducted to identify comorbidities. Any medical diseases were considered if they were listed in the International Classification of Diseases 10th Revision of the International Statistical Classification of Disease and Related Health Problems.

#### Hypertension

Hypertension was defined as a diagnosis before the study started or a systolic blood pressure ≥ 140 mmHg and/or diastolic blood pressure ≥ 90 mmHg during preoperative evaluation [15].

#### Diabetes mellitus

Diabetes was either defined as a diagnosis before the study or based on plasma glucose criteria, either the fasting plasma glucose value or the 2-h plasma glucose value during a 75-g oral glucose tolerance test or the A1C criteria during preoperative evaluation [16].

#### Chronic liver disease

Chronic liver disease was defined as evidence of any of the following in the patient’s chart: (a) at least two of the liver tests (ie, alanine aminotransferase and aspartate aminotransferase) being abnormal, as defined by the laboratory where the test was performed at least 6 months apart; (b) an imaging study with radiological signs of cirrhosis and portal hypertension, or a hepatic mass, and evidence of chronic liver disease; (c) a liver biopsy consistent with chronic liver disease; or (d) a diagnostic clinical event (variceal bleeding, hepatic encephalopathy, spontaneous bacterial peritonitis, ascites) [17].

#### Dementia

Dementia is a syndrome occurring as a result of a brain disease. It consists of impairment of several higher cortical functions, such as memory, comprehension, calculation, learning, judgment, and language [18]. This disease was diagnosed before the study. It includes all kinds of dementia, such as Alzheimer’s disease, vascular dementia, and Parkinson’s disease dementia. Cognitive testing, such as the Mini-Mental State Examination, was conducted in all patients to confirm cognitive impairment.

#### Cerebrovascular accident

Cerebrovascular accident was defined as ischemic stroke, silent infarction, intracerebral hemorrhage, subarachnoid hemorrhage, and silent hemorrhage [19]. Imaging tests, such as magnetic resonance imaging and computed tomography, were previously reviewed to identify lesions in patients with cerebrovascular accidents.

#### Osteoporosis

Osteoporosis was defined as a diagnosis before the study started or bone density 2.5 standard deviations or more below the mean for young normal people during preoperative evaluation [20].

### Functional outcome measures

To evaluate functional outcomes, all patients were assessed immediately after transfer and 6 months post-surgery.

#### Koval’s grade

Koval’s grade is a tool used to evaluate walking dependency. It is classified into seven grades: independent community ambulators (grade 1), community ambulators with a cane (grade 2), community ambulators with walker/crutches (grade 3), independent household ambulators (grade 4), household ambulators with a cane (grade 5), household ambulators with walker/crutches (grade 6), and nonfunctional ambulators (grade 7). A community ambulator refers to a person who can walk indoors or outdoors either independently or with assistive devices. A household ambulator is restricted to walking indoors either independently or with assistive devices. A nonfunctional ambulator refers to a person who is bed-bound or needs help moving from a bed to a chair [21].

#### Functional ambulatory category (FAC)

The FAC is a six-point scale that evaluates functional walking ability. This scale assesses ambulatory ability by determining how much support the patient needs when walking, regardless of whether or not a personal assistive device is used [22]. When evaluating the FAC score, the possibility of walking is based on the ability to walk at least 10 ft. Patients were classified into six grades, ranging from the ability to walk alone anywhere, including stairs (FAC 5), and inability to walk or walk with the help of two or more persons (FAC 0).

#### Functional Independence measures (FIM)-locomotion

The FIM is an 18-item ordinal scale used to evaluate an individual’s physical, psychological, and social function. These 18 items are divided into six domains: self-care, sphincter control, transfers, locomotion, communication, and social cognition. Each of the 18 items is graded on a scale of 1–7 (1 = total assist and 7 = complete independence) [23]. “Locomotion” scale is related to the patient’s ambulation level.

#### Modified Rivermead mobility index (MRMI)

The MRMI uses a six-point ordinal scoring system (0–5 points) to record whether an activity can be performed with the help of two people, one person, supervision, an aid, or independently. The MRMI consists of eight items, each of which includes turning over, lying to sitting, sitting balance, standing up from sitting, standing, transfers, walking indoors, and stair climbing. The total score is 40 points, and a higher score indicates better mobility [24].

#### Berg balance scale (BBS)

The BBS is widely used as a clinical test for an individual’s static and dynamic balance abilities. The items include simple mobility tasks and more difficult tasks. This scale consists of 14 items scored on a scale of 0 to 4. The maximum total score is 56 [25].

#### 4-meter walking speed test (4MWT)

The 4MWT is a simple gait assessment that can be used to assess the usual walking speed. The time taken to walk 4 m was measured with a stopwatch, and walking speed was calculated using this test [26].

#### Korean version of mini-mental state examination (K-MMSE)

The K-MMSE was used to assess cognitive function. It consists of 30 questions (total, 30 points): 10 points for time and place orientation, 3 for memory registration, 3 for memory recall, 5 for attention/calculation, 5 for language, 3 for praxis, and 1 for visuospatial function [27].

#### Geriatric depression scale (GDS)

The GDS is a screening test for depression in older people. This scale comprises 30 simple questions [28].

#### EuroQol five-dimension (EQ-5D) questionnaire

The EQ-5D is an instrument for measuring self-reported general health status. The five dimensions included in the EQ-5D are mobility, self-care, usual activities, pain/discomfort, and anxiety/depression. Each question has three severity levels (no problem, some/moderate problems, and major problems) [29]. The score was converted using utility weights derived from the general Korean population [30]. EQ-5D scores ranged between − 1 and 1 (full health).

#### Korean version of modified Barthel index (K-MBI)

The K-MBI measures the patient’s performance in 10 activities of daily life. The items can be divided into two groups: self-care (feeding, grooming, bathing, dressing, bowel and bladder care, and toilet use) and mobility (ambulation, transfers, and stair climbing). The items are summed to give a score ranging from 0 (completely dependent) to 100 (completely independent) [31].

#### Korean version of instrumental activities of daily living (K-IADL)

The K-IADL is a tool used to evaluate the ability to perform daily activities that allow an individual to live independently in a community. The K-IADL consists of 10 items: decorating, housework, preparing meals, laundry, going out for a short distance, using transportation, shopping, handling money, using a telephone, and taking medicine. Three items (decorating, going out for a short distance and taking medicine) include a three-point scoring system, and 7 items (housework, preparing meals, laundry, using transportation, shopping, handling money and using a telephone) include a four-point scoring system. The average score is measured by dividing the total score by the total number of evaluated questions. The higher the score, the lesser the functionality [32].

#### Korean version of fatigue, resistance, ambulation, illnesses, and loss of weight scale (K-FRAIL)

The FRAIL scale is a screening test for frailty status. This scale consists of five simple questionnaires. A score of 0 was considered robust, 1 to 2 as prefrail, and 3 to 5 as frail [33].

### Statistical analysis

All statistical analyses were performed using Statistical Package for the Social Sciences version 24.0 (IBM, Armonk, NY, USA). A paired t-test was used to compare differences in physical function and quality of life (QoL) immediately after transfer and 6 months post-surgery. A Student’s t-test was performed to compare differences in physical function and QoL according to comorbidities in patients 6 months post-surgery. Multivariable linear regression analysis, adjusted for age, sex, and designated initial values of physical function, QoL, and comorbidities using backward selection, was used to identify factors that showed a significant correlation with physical function and QoL 6 months post-surgery. The significance level of the alpha-value was set at 0.05.

## Results

### Demographic results

The demographic and disease-related characteristics of the patients are shown in Table 1. A total of 211 patients were enrolled in the study. The mean age of all patients was 81.6 ± 6.7 years. Fifty patients were male and 161 were female. The prevalence of hypertension, diabetes mellitus, chronic liver disease, dementia, cerebrovascular accident, and osteoporosis was 64.9% (137), 35.1% (74), 8.5% (18), 9.0% (19), 14.7% (31), and 72.5% (153), respectively.

**Table 1:** Demographics and disease-related characteristics of the subjects (*N* = 211)

Variables	Values
Age (years)	81.6 ± 6.7
Sex, males/females (number)	50 (23.7%) / 161 (76.3%)
Height (cm)	155.7 ± 8.4
Weight (kg)	54.2 ± 9.7
Fracture side (number)	
Right	107 (50.7%)
Left	104 (49.3%)
Fracture site (number)	
Femoral neck	84 (39.8%)
Intertrochanteric	116 (55.0%)
Subtrochanteric	11 (5.2%)
Operation type (number)	
Bipolar hemiarthroplasty	105 (49.8%)
Total hip replacement arthroplasty	12 (5.7%)
Reduction and internal fixation	94 (44.5%)
Time from surgery to RM transfer (days)	7.28 ± 2.8
Hospitalization period at RM (days)	13.6 ± 6.0
ASA PS classification	
Class I	15 (7.1%)
Class II	115 (54.5%)
Class III	71 (33.6%)
Comorbidities (number)	
Hypertension	137 (64.9%)
Diabetes mellitus	74 (35.1%)
Chronic liver disease	18 (8.5%)
Dementia	19 (9.0%)
Cerebrovascular accident	31 (14.7%)
Osteoporosis	153 (72.5%)

Values represent mean ± standard deviation or number (%) of cases

*RM* rehabilitation medicine, *ASA PS* American Society of Anesthesiologists Physical Status

### Evaluation of physical function and QoL immediately after transfer and 6 months post-surgery

Table 2 shows the evaluation of physical function for all patients immediately after transfer and 6 months post-surgery. The scores of all functional outcomes, except MMSE, were improved significantly 6 months post-surgery compared with immediately after the transfer (*p* < 0.001).

**Table 2:** Evaluation of physical function immediately after transfer and 6 months post-surgery (N = 211)

Variable	Values		
	Immediately after transfer	6 months post-surgery	*p*-value
Koval’s grade	6.64 ± 0.50	3.28 ± 1.97	< 0.001
FAC	0.80 ± 1.05	3.56 ± 1.30	< 0.001
FIM-locomotion	1.91 ± 1.39	5.75 ± 1.25	< 0.001
MRMI	17.94 ± 6.67	31.17 ± 11.27	< 0.001
BBS	11.87 ± 12.30	36.69 ± 17.30	< 0.001
4MWT (s)	26.01 ± 15.02	10.15 ± 9.10	< 0.001
K-MMSE	19.58 ± 6.45	19.66 ± 9.35	0.120
GDS	10.86 ± 6.14	6.38 ± 5.90	< 0.001
EQ-5D	0.36 ± 0.17	0.68 ± 0.17	< 0.001
K-MBI	43.58 ± 17.83	77.21 ± 29.95	< 0.001
K-IADL	2.78 ± 0.27	2.03 ± 0.59	< 0.001
K-FRAIL	2.98 ± 0.67	1.74 ± 1.09	< 0.001

Values represent mean ± standard deviation

*FAC* Functional Ambulatory Category, *FIM* Functional Independence Measures, *MRMI* Modified Rivermead Mobility Index, *BBS* Berg Balance Scale, *4MWT* 4-Meter Walking speed Test, *K-MMSE* Korean version of Mini-Mental State Examination, *GDS* Geriatric Depression Scale; *EQ-5D* EuroQol Five-Dimension, *K-MBI* Korean version of Modified Barthel Index, *K-IADL* Korean version of Instrumental Activities of Daily Living, *K-FRAIL* Korean version of Fatigue, Resistance, Ambulation, Illnesses, and Loss of weight scale

### Comparison of physical function and QoL according to comorbidities (Univariate analysis)

Table 3 compares the differences in physical function and QoL according to comorbidities in patients 6 months after surgery. Patients with hypertension had significantly negative K-FRAIL scores (*p* = 0.044). Diabetes mellitus was significantly negatively correlated with the FAC (*p* = 0.004), MRMI (*p* = 0.037), GDS (*p* = 0.036), EQ-5D (*p* = 0.032), K-IADL (*p* = 0.002), and K-FRAIL (*p* = 0.009). Chronic liver disease showed a significant negative correlation with the EQ-5D (0.047). Patients with dementia had significantly negative scores for Koval’s grade (*p* = 0.017), FAC (*p* = 0.013), and K-IADL (*p* = 0.016). Patients with a cerebrovascular disease had lower scores on the MMSE (*p* = 0.019). Osteoporosis was significantly negatively correlated with Koval’s grade (*p* = 0.037), FIM-locomotion (*p* = 0.012), BBS (*p* = 0.035), 4MWT (*p* = 0.001), K-IADL (*p* = 0.002), and K-FRAIL (*p* = 0.043).

**Table 3:** Comparison of physical function and QoL according to comorbidities in patients 6 months after surgery

Variable	Hypertension		Diabetes mellitus		Chronic liver disease	
	(+)	(−)	(+)	(−)	(+)	(−)
Koval’s grade	3.45 ± 2.01	2.96 ± 1.89	3.69 ± 1.94	3.07 ± 1.97	3.43 ± 2.23	3.27 ± 1.97
FAC	3.46 ± 1.25	3.75 ± 1.37	3.14 ± 1.30†	3.78 ± 1.25	3.57 ± 1.81	3.56 ± 1.28
FIM-locomotion	5.79 ± 1.17	5.68 ± 1.40	5.36 ± 1.53	5.89 ± 1.12	5.60 ± 2.07	5.76 ± 1.20
MRMI	31.88 ± 9.95	30.06 ± 13.17	27.27 ± 12.87*	32.69 ± 10.23	33.20 ± 8.70	31.06 ± 11.43
BBS	37.42 ± 15.86	35.53 ± 19.54	31.19 ± 18.80	38.82 ± 16.33	39.00 ± 19.46	36.56 ± 17.28
4MWT (s)	10.10 ± 9.62	10.24 ± 8.29	11.97 ± 13.32	9.58 ± 7.35	18.77 ± 25.22	9.57 ± 6.95
K-MMSE	19.65 ± 8.90	19.67 ± 10.16	17.73 ± 10.17	20.40 ± 8.99	23.40 ± 9.61	19.44 ± 9.35
GDS	6.58 ± 5.99	6.06 ± 5.83	8.48 ± 6.97*	5.60 ± 5.30	10.00 ± 4.58	6.17 ± 5.92
EQ-5D	0.66 ± 0.17	0.70 ± 0.19	0.63 ± 0.22*	0.70 ± 0.15	0.54 ± 0.21*	0.68 ± 0.18
K-MBI	80.03 ± 26.50	72.70 ± 34.66	68.43 ± 34.61	80.82 ± 27.26	83.00 ± 25.58	76.89 ± 30.26
K-IADL	2.09 ± 0.58	1.93 ± 0.60	2.24 ± 0.53†	1.93 ± 0.59	2.00 ± 0.79	2.04 ± 0.58
K-FRAIL	1.87 ± 1.07*	1.49 ± 1.08	2.06 ± 1.07†	1.57 ± 1.07	1.86 ± 1.07	1.73 ± 1.09
Variable	Dementia		Cerebrovascular accident		Osteoporosis	
	(+)	(−)	(+)	(−)	(+)	(−)
Koval’s grade	4.58 ± 2.20*	3.17 ± 1.92	3.36 ± 2.17	3.27 ± 1.95	3.48 ± 1.95*	2.72 ± 1.96
FAC	2.67 ± 1.61*	3.64 ± 1.24	3.41 ± 1.37	3.58 ± 1.29	3.44 ± 1.28	3.90 ± 1.31
FIM-locomotion	5.50 ± 1.38	5.77 ± 1.25	5.29 ± 1.60	5.79 ± 1.22	5.54 ± 1.34*	6.30 ± 0.77
MRMI	28.71 ± 13.19	31.37 ± 11.17	24.56 ± 15.87	31.88 ± 10.55	30.64 ± 10.92	32.54 ± 12.24
BBS	33.00 ± 20.00	36.99 ± 17.16	23.67 ± 24.02	38.08 ± 15.99	34.34 ± 17.01*	42.73 ± 16.88
4MWT (s)	10.25 ± 6.70	10.15 ± 9.31	9.24 ± 7.66	10.22 ± 9.23	11.57 ± 10.26†	6.47 ± 2.70
K-MMSE	15.43 ± 10.06	20.00 ± 9.27	12.78 ± 10.07*	20.39 ± 9.03	19.12 ± 9.48	21.04 ± 9.06
GDS	7.50 ± 5.82	6.30 ± 5.93	6.00 ± 7.07	6.42 ± 5.83	6.92 ± 5.96	5.00 ± 5.61
EQ-5D	0.58 ± 0.28	0.68 ± 0.17	0.67 ± 0.17	0.68 ± 0.18	0.67 ± 0.17	0.69 ± 0.21
K-MBI	65.86 ± 37.38	78.10 ± 29.36	59.33 ± 39.93	79.06 ± 28.38	74.49 ± 29.45	84.15 ± 30.63
K-IADL	2.43 ± 0.56*	2.00 ± 0.58	2.05 ± 0.69	2.03 ± 0.57	2.12 ± 0.55†	1.78 ± 0.61
K-FRAIL	2.25 ± 1.29	1.69 ± 1.06	1.68 ± 1.21	1.75 ± 1.07	1.85 ± 1.11*	1.44 ± 0.97

Values represent mean ± standard deviation

*FAC* Functional Ambulatory Category, *FIM* Functional Independence Measures, *MRMI* Modified Rivermead Mobility Index, *BBS* Berg Balance Scale, *4MWT* 4-Meter Walking speed Test, *K-MMSE* Korean version of Mini-Mental State Examination, *GDS* Geriatric Depression Scale, *EQ-5D* EuroQol Five-Dimension, *K-MBI* Korean version of Modified Barthel Index, *K-IADL* Korean version of Instrumental Activities of Daily Living, *K-FRAIL* Korean version of Fatigue, Resistance, Ambulation, Illnesses, and Loss of weight scale

* *p* < 0.05, † *p* < 0.01

### Comorbidities influencing functional outcome 6 months after fragility hip fracture surgeries

The results of the multiple regression analysis are summarized in Table 4. Multivariable linear regression analyses revealed that the functional outcomes of Koval’s grade (ß = 0.158; *p* = 0.038) and K-FRAIL (ß = 0.205; *p* = 0.010) 6 months after fragility hip fractures were significantly affected by dementia. In addition, the FAC (ß = − 0.185; *p* = 0.016), GDS (ß = 0.227; p = 0.038), EQ-5D (ß = -0.158; *p* = 0.047), K-IADL (ß = 0.170; p = 0.01), and K-FRAIL (ß = 0.184; *p* = 0.018) were significantly associated with diabetes mellitus at 6 months. In addition, FIM-locomotion (ß = − 0.215; *p* = 0.048) was significantly correlated with osteoporosis, BBS with cerebrovascular accident (ß = -0.270; *p* = 0.008), and K-FRAIL with hypertension (ß = 0.207; p = 0.008) 6 months after fragility hip fractures.

**Table 4:** Predictors of functional outcomes 6 months after fragility hip fracture surgeries

Functional outcome/independent predictor (comorbidity)	Standardized (ß)	*p*-value	Adjusted R^2^
Koval’s grade			0.178
Initial Koval’s grade	0.344	< 0.001	
Dementia	0.158	0.038	
FAC			0.199
Initial FAC	0.178	0.023	
Diabetes mellitus	-0.185	0.016	
FIM-locomotion			0.109
Initial FIM-locomotion	0.249	0.023	
Osteoporosis	-0.215	0.048	
BBS			0.126
Age	−0.232	0.023	
Cerebrovascular accident	−0.270	0.008	
GDS			0.128
Initial GDS	0.304	0.006	
Diabetes mellitus	0.227	0.038	
EQ-5D			0.096
Initial EQ-5D	0.224	0.006	
Diabetes mellitus	−0.158	0.047	
K-IADL			0.391
Age	0.216	0.003	
Initial K-IADL	0.436	< 0.001	
Diabetes mellitus	0.170	0.010	
K-FRAIL			0.160
Hypertension	0.207	0.008	
Diabetes mellitus	0.184	0.018	
Dementia	0.205	0.010	

Multivariable regression analyses adjusted for age, sex, initial functional outcome variables, and other comorbidities

*FAC* Functional Ambulatory Category, *FIM* Functional Independence Measures, *BBS* Berg Balance Scale, *GDS* Geriatric Depression Scale, *EQ-5D* EuroQol Five-Dimension, *K-IADL* Korean version of Instrumental Activities of Daily Living, *K-FRAIL* Korean version of Fatigue, Resistance, Ambulation, Illnesses, and Loss of weight scale

## Discussion

This study demonstrates that numerous comorbidities significantly influence physical function outcomes and QoL 6 months after fragility hip fracture surgeries. First of all, the association between dementia and functional outcomes in patients with fragility hip fractures is controversial. In this study, Koval’s grade related to walking dependency scored higher in patients with dementia at 6 months post-surgery, meaning that patients with dementia had less gait recovery than those without dementia. This finding is similar to that by Lenze et al. [34] who used the motor scale of the FIM. They concluded that cognitive impairment negatively affects rehabilitation outcomes in older patients with hip fractures. They argued that the poor rehabilitation outcome of inpatients is mediated by participation in rehabilitation. In addition, some studies have reported that only severe cognitive impairment is associated with poor functional outcomes. Using the Activities of Daily Living index, Soderqvist et al. [35] demonstrated that severe cognitive impairment has a poor effect on walking ability, ability to perform activities of daily living, and mortality after hip fractures. They argued that cognitive impairment is a major risk factor in choosing surgical methods and planning postoperative care, and that these limited choices lead to decline in functional outcomes. However, some studies have stated that cognitive impairment itself is not related to functional results related to muscle strength or activities of daily living in patients with fragility hip fractures [36, 37]. Beloosesky et al. [37] concluded that patients with fragility hip fractures with cognitive impairment can obtain the same motor functional outcome as those with normal cognition if they were mobile pre-fracture. The effect of dementia on frailty in cases of fragility hip fractures has not been described in the literature. However, many studies have reported an association between dementia and frailty. Raji et al. [38] showed that initially, non-frail older patients with poor cognition have a higher risk of becoming frail over 10 years than those with normal cognition, regardless of other demographic and health factors. They argued that cognitive impairment may be associated with under-recognition of risk factors for frailty, such as poor exercise participation and poor nutrition intake, and undertreated comorbidities, which are known to affect frailty. According to this hypothesis, the negative effect of dementia on the K-FRAIL score in our study can be considered to be due to the persistence of the risk factors and comorbidities.

When it comes to the relationship between diabetes mellitus and fragility hip fractures, patients with diabetes mellitus are well known to have a higher risk of developing fragility hip fractures [39]. However, studies on the effects of diabetes mellitus on functional outcomes post-surgery in patients with fragility hip fractures are limited. Lieberman et al. [40] compared rehabilitation outcomes between diabetic and non-diabetic patients among older patients with hip fractures using the FIM scale. They concluded that the rehabilitation outcome of diabetic patients was significantly worse than that of non-diabetic patients. In contrast, Tian et al. [41] showed that diabetes does not affect post-fragility hip fracture functional outcomes. However, they also reported the occurrence of more postoperative complications, such as urinary tract infections and deep vein thrombosis, in patients with diabetes. In our study, we found that diabetes affected a variety of functional outcomes, including the FAC related to gait function, GDS related to depression, EQ-5D related to comprehensive QoL, IADL related to self-reliance, and K-FRAIL related to frailty. Postoperative complications, which are more likely to occur in patients with diabetes, can be assumed to affect many aspects of functional damage. In general, many postoperative complications occur well in patients with diabetes. This may result in inadequate rehabilitation or additional damage. Therefore, further studies are needed to determine the relationship between postoperative complications and several functional outcomes in patients with fragility hip fractures and diabetes mellitus.

Osteoporosis is a well-known risk factor for hip fracture, but no literature has shown that osteoporosis affects functional outcomes in patients with fragility hip fractures. Since osteoporosis is not a symptomatic disease and is a systemic disease characterized by low bone mineral density, it is not reasonable that osteoporosis itself negatively affects the functional outcomes of fragility hip fractures in the absence of other factors. However, Makridis et al. [42] showed that osteoporotic treatment can positively affect functional recovery, re-fracture rate, QoL, and hardware-related complications in patients with hip fractures.

In this study, cerebrovascular accidents had a negative effect on the BBS, a functional evaluation tool related to gait. In the analysis by Penrod et al. [43], cerebrovascular accidents reduced independent mobility and ADLs 6 months after fragility hip fractures. They explained that these functional impacts of cerebrovascular accidents may be due to the direct and consistent effects of these conditions on mobility and strength. In addition, Mathew et al. [6] found that older hip fracture patients with stroke have a worse functional improvement 1 year after a fracture than those without stroke.

No direct studies have been conducted on the relationship between hypertension and frailty in patients with fragility hip fractures, but the association between hypertension and frailty has been studied extensively [44–46]. In this regard, Kang et al. [44] explained that uncontrolled hypertension can cause serious cardiovascular events and hypertension is related to future ADL/IADL limitation or disability.

There are also previous studies of risk factors for poor functional recovery in other major fragility fractures, such as vertebral and proximal humerus [47, 48]. Especially, Chao et al. [47] investigated the factors affecting the functional outcome at 4 months in hip fracture patients and vertebral fracture patients. They confirmed that comorbidities (including chronic kidney disease, heart and neurologic diseases, cancer, osteoarthritis, and diabetes) negatively affected short-term functional recovery. This suggests that comorbidities can also have a great influence on functional recovery in fragility fractures not only in the hip but also in other areas.

Patients with fragility hip fracture require a multidisciplinary and complex management to deal with many problems, including comorbidities. Considering the complexity of the patient with fragility hip fracture, co-management with several departments has recently been proposed. De Vincentis et al. [49] presented an Italian consensus on the management of patients with hip fracture. They discussed several topics related to hip fracture patients (including optimal care path, management of comorbidities, screening and correction of risk factors for hip fracture, etc.), and emphasized co-shared management of orthopedics and geriatrics.

Our study has several limitations. First, the severity of the comorbid disease state is difficult to assess, making it difficult to determine the functional outcome according to the severity of the comorbid disease. The American Society of Anesthesiologists Physical Status (ASA PS) classification was additionally described to complement a patient’s general medical condition. Second, because the study was conducted on patients who received rehabilitation treatment at a tertiary hospital, the study population may not represent the general community-dwelling older adults who have suffered from fragility hip fractures. Third, the pre-fracture functional status was not evaluated in this study. Since preoperative functional status is likely to be poor in patients with comorbidities, further studies are needed to evaluate the effect of this on postoperative functional outcomes. Finally, the total number of patients was large, but the number of patients with comorbidities other than hypertension, diabetes mellitus, and osteoporosis was relatively small. In other words, statistical data about chronic liver disease, dementia and cerebrovascular accident were based on small groups and require further investigation. In addition, among the numerous comorbidities, only comorbidities with a relatively high frequency could be studied. In this study, the total number of cancer and cardiac disease cases was also examined, but the number of cases of sub-diseases of the two diseases was too small. In addition, these diseases are heterogeneous and have a large difference in severity; thus, these diseases could not be included in the statistical analysis. Therefore, larger studies are needed to evaluate the effects of more diverse comorbidities on the functional outcome post-fragility hip fracture surgeries.

Despite these limitations, this study can contribute in numerous ways to the functional results after fragility hip fracture surgeries. First, the literature shows which comorbidity can affect the postoperative functional outcomes; thus, clinicians can determine which comorbidity should be intensively controlled in patients with fragility hip fractures. Second, clinicians can predict and prepare for poor functional outcomes when a patient has certain comorbidities. Specifically, identifying the risk factors for the poor functional outcomes can provide focused intervention strategies for those with the highest likelihood of poor outcomes. Finally, in contrast to other studies, we investigated the effects of several diseases at once, enrolled a greater number of patients (*n* = 211), and used a variety of functional outcome tools.

## Conclusion

This study confirmed that comorbidities, particularly dementia and diabetes mellitus, significantly influenced functional outcomes with regard to several performance-based physical function tests and QoL 6 months after fragility hip fracture surgeries.

## Data Availability

The datasets used and analyzed during the current study are available from the corresponding author on reasonable request.

## Abbreviations

FAC: Functional Ambulatory Category
FIM: Functional Independence Measure
MRMI: Modified Rivermead Mobility Index
BBS: Berg Balance Scale
4MWT: 4-Meter Walking speed Test
K-MMSE: Korean version of Mini-Mental State Examination
GDS: Geriatric Depression Scale
EQ-5D: EuroQol Five-Dimension
K-MBI: Korean version of Modified Barthel Index
K-IADL: Korean version of Instrumental Activities of Daily Living
K-FRAIL: Korean version of Fatigue, Resistance, Ambulation, Illnesses, and Loss of weight scale
QoL: Quality of life
ASA PS: American Society of Anesthesiologists Physical Status
